# Evaluation of Factors Associated with Sexual Function in Infertile Women 

**DOI:** 10.22074/ijfs.2018.5193

**Published:** 2018-03-18

**Authors:** Somayeh Alirezaei, Gity Ozgoli, Hamidreza Alavi Majd

**Affiliations:** 1 Student Research Committee, Department of Midwifery, School of Nursing and Midwifery, Mashhad Univer- sity of Medical Sciences, Mashhad, Iran; 2 Department of Midwifery and Reproductive Health, Faculty of Nursing and Midwifery, Shahid Beheshti University of Medical Sciences, Tehran, Iran

**Keywords:** Female, Infertility, Self-Efficacy, Sexual Behavior

## Abstract

**Background:**

Infertility is a major and problem influencing different aspects of couples life, especially those of women. Sexual dysfunction is the silent partner of infertility. This study aimed to identify the above-mentioned factors to make necessary decisions and perform efficient interventions to improve the sexual health of infertile women. This study investigated the factors influencing sexual dysfunction in infertile women in Mashhad, Iran.

**Materials and Methods:**

This cross-sectional study was conducted on 85 infertile women visiting governmental Infertility Clinic and Research Center in Mashhad, Iran. The convenience sampling method was used in this study. The research tools included a demographic and infertility information form, a sexual self-efficacy questionnaire based on Schwarzer’s General Self-Efficacy Scale, Female Sexual Function Index (FSFI), and Evaluation and Nurturing Relationship Issues, Communication, and Happiness (ENRICH) Marital Satisfaction Scale. The descriptive statistical tests and logistic regression method were used to analyze data.

**Results:**

The mean age of women was 31.18 ± 5.56 years old. The majority of participants (36.7%) had higher educa- tions, and 60% of them were housewives. Most of their husbands (49.4%) were self-employed. The mean period of infertility awareness was 6.02 ± 4.47 years, and the mean period of infertility treatment was 4.11 ± 4.46 years. The following variables influenced the sexual function of infertile women: sexual self-efficacy, sexual satisfaction, marital satisfaction, the educational level of both wife and husband, income, satisfaction with spouse appearance, and the high costs of infertility treatment.

**Conclusion:**

The findings indicated that some factors such as sexual self-efficacy, marital satisfaction, sexual satisfac- tion, education, and cost of infertility treatment are associated with sexual function in infertile women.

## Introduction

Infertility is be defined as being unable to become pregnant after having regular sexual contacts for one year without using contraceptive methods (1, 2). Infertility is a health issue in today’s world. According to the statistics published by the World Health Organization on January 16, 2013, in developed countries one out of every four couples is suffering from infertility (3). In general, male factor, female factor, joint factor and unknown factor account for 30-40%, 40-50%, 10-20% and 5-10% of infertility cases, respectively (4). Infertility is observed in 10- 15% of American couples (5).

Several studies have been conducted on infertile couples in Iran. In an epidemiological study in 2004, the prevalence of infertility was reported 24.9% in Iranian women of 19 to 49 years old (6). According to the statistics presented by Iran’s Ministry of Health and Medical Education in 2009, the prevalence of infertility was estimated to be 20.2% in Iran (7). Sexual dysfunctions comprise a heterogeneous group of disorders that are typically characterized by a clinically significant disturbance in a person’s ability to respond sexually or to experience sexual pleasure (8). Sexual problems are highly prevalent in women. In the United States, approximately 40% of women have sexual concerns and 12% report distressing sexual problems. Female sexual dysfunction takes different forms, including lack of sexual desire, impaired arousal, inability to achieve orgasm, pain with sexual activity, or a combination of these issues (9).

The most important causes of sexual dysfunction in women include lack of desire, excitement disorders, lack of orgasm, dyspareunia and vaginism. The prevalence rates of sexual dysfunction are 40 and 19.6% in the US and Sweden respectively (2). Since an active and effective sexual contact can increase the chance of fertility, it is believed that sexual dysfunction has a more marked impact on infertile women rather than fertile women (10). Sexual dysfunction is associated with infertility. It is caused by the reduction or lack of sexual activity. Sexual dysfunction is usually created or intensified during the diagnosis and treatment of infertility. In fact, sexual dysfunction is the silent partner of infertility. It prevents effectiveness treatment of infertility (11).

Sexual behavior disorders can be a prior cause of infertility. Generally, infertile couples seek treatment for infertility instead of searching for deeper problem in their sexual relationships (12). It was reported that many infertile women suffer from one of the different types of sexual dysfunction (13). Many factors can influence sexual dysfunction. The physical factors include the ages of husband and wife, BMI, physical activities, etc. The mental-emotional factors include feelings for a sexual partner, sexual self-efficacy, self-confidence, the mental image of body, the feeling of sexual attraction, etc. Finally, the social factors include the educational levels of a women and her husband, occupation, duration of marriage, the quality of spousal relationships, socioeconomic status, substances abuse, etc. (14). Many couples believe that conception is the only result of a sexual relationships; otherwise, this relationship is fruitless (15).

Since fertility is regarded as a value in Iranian culture and notions, infertility questions the individual and social competencies of women, i.e. the feelings of maternity and spousal values); therefore, a women’s entire marital and sexual relationships with her husband will be affected. Given the importance of identifying the factors influencing sexual dysfunction in infertile women, the current study employed a modern approach using statistical models to investigate the subject. This study aimed to identify the above-mentioned factors to make necessary decisions and perform efficient interventions to improve the sexual health of infertile women. This study investigated the factors influencing sexual dysfunction in infertile women in Mashhad, Iran.

## Materials and Methods

In this cross-sectional study, type I error (Za=1.96) and the statistical power (Zß=1.28) were taken into account to select 85 infertile women as the population sample. The following formula was used for sampling: 

$$n\geq 2\frac{{\left(Z_{\alpha }+Z_{\beta }\right)}^{2}\sigma^{2}}{{\left({µ}_{1}-{µ}_{2}\right)}^{2}}$$

The convenience sampling method was employed to select the participants from the women visiting Montasarieh Infertility Clinic and Research Center in Mashhad, Iran. The Ethics Committee of Shahid Beheshti University of Medical Sciences approved this study (approval No. 2881/116). All participants received information about the purpose of this study and gave their verbal informed consent to participate. 

The participants were aged between 20 and 45 years old. Participants, did not suffer from any physical, mental, or medical problems. They were not addicted to any substances or alcohol. The information collection tools included an informational form with demographic and infertility information and Female Sexual Function Index (FSFI) and at demographic variables included age, mental and physical status, surgery history, medication and drugs, having a child or foster child. Infertility variables were duration of infertility and treatment, cause of infertility, kind of treatment, hope for cure, cost of treatment, and information about sexual relationship sexual. FSFI is one of the gold standard for female sexual dysfunction (FSD) assessment and in this study, the nominal and content validities of FSFI were re-evaluated by obstetricians and gynecologists, psychologists, midwives, and health education experts. 

The reliability of this index was determined 0.9 through a retest. The researcher obtained the information from participants individually. The standard FSFI includes 19 items regarding sexual desire, sexual excitement, dyspareunia, and the inability to reach orgasm. Each item has five choices. According to the scoring system, a score below 28 indicates a poor sexual function, whereas a score between 28 and 36 shows a desirable sexual function (9). Sexual self-efficacy questionnaire developed based on Schwarzer’s General Self-Efficacy Scale includes 10 items. Each item has three choices. Following completion of the questionnaire, a score of -0-10- was considered low, -10-20- moderate and -20-30- high efficacy (16). 

ENRICH Marital Satisfaction Scale includes 47 items regarding idealistic distortion, marital satisfaction, personality issues, communication, conflict resolution, etc & with 5-Likert scale. According to the scoring system, a score below 30 indicates very poor satisfaction, -30-40- poor satisfaction, -40-60- moderate satisfaction, -60-70- high satisfaction and above 70 very high satisfaction (17). Then the descriptive- analytical tests were used to analyze the results. Finally, the logistic regression method was employed to investigate the relation between influencing and predicting factors and sexual function in infertile women, because sexual function possesses qualitative variables with two state (undesirable=0 when sexual function less than 28 according FSFI and desirable= 1 when sexual function is between 36-28). 

## Results

Infertile women were investigated with respect to demographic characteristics (Table 1). The mean age of infertile women was 31.18 ± 5.56 years old, and 32% of them were aged between 26 and 30 years old. The cause of infertility was the female factor in 28 participants (32.9%), the male factor in 24 participants (28.3%), joint factor in 15 participants (17.6%), and unknown factor in 15 participants (17.6%). Moreover, three of them (3.5%) visited the clinic for the first time. The average duration of diagnosed infertility was 6.02 ± 4.47 years, and the average duration of infertility treatment was 4.11 ± 4.41. Furthermore, 54.1% of infertile women reported high levels of marital satisfaction, and 60% of them (51 participants) reported medium self-efficacy in their sexual relationships. 71.8% of infertile women show poor sexual performances (Table 2). 

**Table 1:** Distribution of some demographic variables among infertile women


Variable		n (%)

Education		
	Primary education	23 (34.0)
	High school	25 (29.4)
	Higher education	31 (36.7)
Husband education	
	Primary education	31 (36.55)
	High school	26 (30.6)
	Higher education	28 (32.9)
Job	
	Housewife	60 (70.6)
	Practitioner	25 (29.4)
Income	
	Less than 1,700,000,0 Rials	22 (25.9)
	Equal to 1,700,000,0 Rials	62 (72.9)
	More than 1,700,000,0 Rials	1 (1.2)


**Table 2:** "Number and percent" of sexual function among infertile women


Variable	n (%) or mean + SD

Sexual function desirable,	24 (28.2)
Sexual function undesirable	61 (71.8)
Total score of sexual function	25.93 + 4.32


Table 3 indicated some infertility information. Based on the logistic regression model, the following variables influenced social function: sexual self-efficacy, sexual satisfaction, marital satisfaction, couple satisfaction with spouse appearance, and the high costs of infertility treatment (Table 4).

**Table 3:** Distribution of some infertility information


Variable		n(%)

Using of ART		
	Yes	67(78.8)
	No	18(21.2)
ART IUI		ART
	IUI	25(30.6)
	IVF	21(24.7)
	Ovulation and intercourse	20(23.5)
	None	18(21.2)
Hope for a cure		
	Yes	57(67)
	Somewhat	26(30.6)
	No	2(2.4)
The cost problem	
	Yes	58(68.2)
	Somewhat	21(24.7)
	No	6(7.1)


ART; Assisted reproductive technology, IUI; Intrauterine inseminations, and IVF; *In vitro* fertilization.

**Table 4:** Regression logistic of related factors of sexual function


Related factor	OR (CI)	P value

low	13.77 (1.74-10.89)	0.01
Average	4.16 (2.35-7.33)	0.00
High	1	-
No	3.78 (2.19-6.51)	0.00
Yes	1	-
Sexual satisfaction		
No	5.52 (2.66-11.43)	0.00
Yes	1	-
Primary education	3.42 (1.70-6.94)	0.00
High school	1.89 (1.04-3.41)	0.00
After high school	1	-
Primary education	2.95 (1.40-6.20)	0.01
High school	3.12 (1.73-5.61)	0.00
After high school	1	-
Less than 1,700,000,0 Rials	10.31 (3.03-34.99)	0.00
Equal to 1,700,000,0 Rials	4.20 (1.56-11.31)	0.00
More than 1,700.000.0 Rials	-	-
No	5.14 (1.50-17.64)	0.00
Yes	1	-
No	4.67 (1.75-12.44)	0.00
Yes	1	
Yes	2.66 (0.39-17.91)	0.31
Somewhat	8.54 (1.38-52.73)	0.01
No	1	


OR; Odds ratio and CI; Confidence interval

## Discussion

This research indicated that 71.8% of infertile women were suffering from sexual dysfunction. Regardless of the duration and causes, infertility causes mental health and sexual problems and infertile women suffer from these conditions more than fertile women (16). Previous studies indicate that sexual self-efficacy is related to sexual function, as individuals with a poor sexual self-efficacy and individuals with average sexual self-efficacy face problems in sexual function fourteen times and five times more than individuals with high sexual self-efficacy, respectively.

In 2013, Champion and et al. (17) conducted a study entitled sexual self-efficacy and marital satisfaction on 194 university students. They showed that sexual satisfaction referred to an individual’s pleasure from the type of sexual relationships. Also, the concept of marital-sexual satisfaction depends on an individual’s perception of self-efficacy whether as sexual activity satisfaction or emotional satisfaction. According to another study, self-efficacy and self- confidence should increase in sexual issues to have better and healthier sexual functions (18). Previous investigations indicated that marital satisfaction was significantly related to sexual function. In other words, sexual satisfaction can result in fewer complaints made by women with sexual disorders (19). In fact, a sexual partner’s behavior, sexual adequacy, and martial life status influence sexual function. 

Women having happy and exciting relationships with their husbands experience sexual disorders less often. They feel more self-confident and think that their husbands like their bodies; therefore, they feel that they are more sexually attractive to their husbands. On the other hand, women having negative attitudes towards their bodies are nervous in private and romantic relationships with their husbands. Such women are not sure about having sexual activities (20-22). The results of this study indicate that satisfaction with spouse appearance improves sexual function by five times in both men and women. Likewise, Kalra et al. (23) stated that the shape of body is one of the factors influencing the emergence of sexual dysfunction. Moreover, this study indicated a relation between educational attainment and sexual function. As individuals with high school diplomas or lower educations face sexual dysfunction problems three times more than the individuals with higher educations. 

Fajewonyomi et al. (24) indicated that women with higher educations would face sexual dysfunction less often. In fact, higher educational attainments increase the chance that individuals can speak about their sexual problems or their spouses. Training and high educational attainments are necessary to have desirable and normal sexual activities (25).

Income is another effective factor (22). To confirm this statement, the results of this study showed that low-income individuals face sexual problems four times more than high-income individuals. Moreover, Audu (26) indicated that income would influence sexual function. Also, Cayan et al. (27) suggested low income as a risk factor for the emergence of sexual dysfunctions. Difficulty in providing the costs of infertility treatment increases the chance of sexual dysfunction by nine times. Similarly, Mollaiy nezhad et al. (28) stated that sexual dysfunction was significantly related to the duration of infertility treatment, treatment costs, the number of unsuccessful pregnancies, and the hope for successful treatment. Likewise, Noorani et al. (29) and Karamidehkordi and Latifnejad Roudsari (30) showed that infertile women whose husbands helped them during treatment and covered the costs, experienced better marital and sexual satisfaction. One of our research limitations was inclusion of women with primary infertility. In addition, the infertile women were selected from only one infertility clinic in Mashhad, we used self-report scales and clinician rated psychological parameters.

## Conclusion

The finding indicated that some factors such as sexual self-efficacy, marital satisfaction, sexual satisfaction, education, cost of infertility treatment are associated with sexual function in infertile women. 
